# ASSOCIATION OF RESTRICTED LIFE SPACE MOBILITY WITH COGNITIVE FUNCTION WITHIN OLDER BLACKS AND WHITES WITH DIABETES

**DOI:** 10.1093/geroni/igz038.1925

**Published:** 2019-11-08

**Authors:** Olivio J Clay, Pamela Bowen, Loretta Lee, Gina McCaskill, Olivia Affuso, Michael Crowe

**Affiliations:** University of Alabama at Birmingham, Birmingham, Alabama, United States

## Abstract

The Centers for Disease Control and Prevention have reported that approximately one of every eight older adults self-report experiencing confusion or memory loss that is becoming more frequent or getting worse. Thus, identifying individuals who are at-risk for cognitive problems is essential. The purpose of this investigation was to assess the relationship between life space mobility and cognition within older Blacks and Whites with diabetes. Baseline data from the University of Alabama at Birmingham (UAB) Diabetes and Aging Study of Health (DASH) were utilized. Multiple regression models adjusted for age, education, income, gender, and race were utilized to assess the association between restricted life space (a score of less than 60 on the UAB Life Space Assessment) and cognitive function as assessed by the Telephone Interview for Cognitive Status (TICS-M). The analytic sample consisted of 224 older adults with diabetes (mean age = 73.52) with 54% being female and 53% White. Of the participants, 75 (32%) had a restricted life space and individuals with restricted life space on average had cognition scores that were over 2 points lower than participants categorized as not having restricted life space (B = -0.18, p < .01). Additionally, Black participants had lower levels of cognition when compared to Whites in the covariate-adjusted models (B = -0.23, p < .01). Results of this investigation provide additional evidence to support the relationship between mobility and cognition. Longitudinal investigations assessing the association between mobility and cognition within older adults with diabetes are needed.

